# New squatting test indices are useful for assessing baroreflex sensitivity in diabetes mellitus

**DOI:** 10.1111/j.1464-5491.2008.02591.x

**Published:** 2008-11

**Authors:** M Nakagawa, T Shinohara, F Anan, K Yufu, N Takahashi, N Okada, M Hara, H Yoshimatsu, T Saikawa

**Affiliations:** Division of Laboratory Medicine, Department of Cardiovascular Science, Oita University School of MedicineOita, Japan; * Division of Internal Medicine, Department of Cardiovascular Science, Oita University School of MedicineOita, Japan

**Keywords:** autonomic neuropathy, baroreflex sensitivity, diabetes, squatting test

## Abstract

**Aims:**

The heart rate (HR) responses after performance of the squatting and standing manoeuvre are thought to be a useful tool to assess autonomic neuropathy in diabetics. Our aim was to develop new simple squatting test indices and to analyse their applicability to the assessment of baroreflex sensitivity (BRS) in patients with diabetes.

**Methods:**

Twenty healthy volunteers (mean age 23.2 ± 3.8 years) and 51 patients with diabetes (mean age 55.9 ± 10.6 years) were enrolled in study 1 and study 2, respectively. Each subject stood for 3 min (basal period), then squatted down for 1 min (Sq) and stood up again for 1 min (St). In study 1, the squatting test was performed before and after pharmacological autonomic blockade. In study 2, we measured HR in each period and calculated the difference between basal HR and HRSq (ΔHRSq) and between HRSt and HRSq (ΔHRSt). BRS was also measured using the phenylephrine method in diabetic patients.

**Results:**

In healthy individuals during autonomic blockade, HR changes were mainly controlled by the vagal tone during squatting and by the sympathetic tone during standing. In diabetic patients, ΔHRSq and ΔHRSt positively correlated (*r* = 0.86, *P* < 0.0001) and both ΔHRSq and ΔHRSt significantly correlated with BRS (*r* = 0.66, *P* < 0.0001 and *r* = 0.61, *P* < 0.0001, respectively).

**Conclusions:**

The new squatting test indices provide useful information for assessing autonomic neuropathy and for identifying diabetic patients at high risk of cardiovascular events.

## Introduction

Autonomic neuropathy, a common complication of diabetes mellitus, is associated with increased mortality [1,2]. Baroreflex sensitivity (BRS) is a marker for the ability to augment vagal activity. BRS is attenuated in diabetic patients with autonomic neuropathy [3–6] and is associated with cardiovascular mortality [7] or sudden cardiac death [8].

We previously reported a new physiological, non-invasive method to measure baroreflex response during downward tilting [9,10]. We demonstrated a strong correlation between the systolic blood pressure increase and a corresponding lengthening of the RR interval during downward tilting and a significant correlation with the BRS value in diabetic patients undergoing the conventional phenylephrine method [10]. However, this method is not available for routine screening tests because it requires an electrical tilt table and equipment for continuous blood pressure monitoring.

Simple posture changes such as squatting from standing are followed by a cardiovascular response similar to that seen during downward tilting. According to Marfella *et al*. [11], the heart rate (HR) responses observed after squatting and standing (squatting test) are useful for assessing autonomic neuropathy in diabetic patients. Using their method, we found that squatting test results correlated significantly with BRS assessed by the conventional method in diabetes [12]. However, as beat-to-beat RR intervals must be continuously measured to calculate the squatting test indices, the usefulness of the conventional method in clinical practice is limited. The most important factors for clinically widespread testing are their simplicity and their time- and cost-effectiveness.

Here we report simple squatting test indices acquired with a digital electronic sphygmomanometer, an instrument that is now available in most clinics. We measured the HR responses to the squatting test in healthy subjects under pharmacological autonomic blockade and analysed the autonomic mechanisms involved. We then considered the implications of these new indices for assessing BRS in diabetic patients.

## Subjects and methods

### Study population

We enrolled 20 healthy volunteers (nine men and 11 women) aged 18 to 32 years (mean 23.2 ± 3.8 years) in study 1 and 51 patients with Type 2 diabetes mellitus (28 men and 23 women) aged 30 to 79 years (mean 55.9 ± 10.6 years) in study 2.

Study 1 was designed to assess the role of autonomic involvement in the squatting test in 20 healthy volunteers. They had no relevant medical history and their blood pressure, electrocardiograms, echocardiograms and chest X-rays were normal. None of them took any medication.

In study 2 we investigated the applicability of our squatting test indices on BRS in 51 patients with diabetes mellitus. The consecutive patients who were admitted to the diabetes clinic of our department from April 2003 until March 2004 were enrolled in this study. Patients with atrial fibrillation, frequent atrial or ventricular arrhythmias or atrioventricular block were excluded from the study, as were patients with clinical evidence of heart or renal failure, peripheral vascular disease or a history of myocardial infarction or cerebrovascular disease. Patients who had to discontinue the squatting test as a result of extreme obesity or trouble with their knee joints were also excluded. The patients had a mean duration of diabetes of 8.2 ± 7.1 years (range 1–14) and mean glycated haemoglobin (HbA_1c_) 7.8 ± 1.5%. Nineteen of the 51 patients had clinical evidence of autonomic neuropathy. Eleven patients received insulin and 22 patients oral glucose-lowering agents including sulfonylurea (*n* = 13), alpha glucosidase inhibitors (*n* = 9), pioglitazone (*n* = 1) and metformin (*n* = 2). The remaining patients (*n* = 18) were treated with dietary therapy only. None of the patients was taking any medication known to affect the autonomic nervous system, such as β-blockers. Written informed consent was obtained from all study subjects and the study was approved by our institutional review board.

### Study design

#### Study 1

The 20 healthy subjects underwent the squatting tests before and after pharmacological autonomic blockade. The study was performed in the morning (09.00–11.00 h) under standardized conditions in a quiet room at a comfortable temperature. All had fasted for at least 2 h before testing and they had been asked not to smoke or drink beverages containing alcohol or caffeine for at least 12 h before the study. With the subject supine on a bed, the digital electronic sphygmomanometer (STBP-780B; Colin, Komaki, Japan) was placed over the brachial artery at roughly the vertical level of the heart. The device was an auscultatory unit using R-wave gating in the identification of Korotkoff sounds. Standard 12-lead electrocardigrams (ECGs) were continuously monitored and data were stored in a PCM data recorder (RD-200T; TEAC, Tokyo, Japan) for subsequent analysis. A venous catheter was placed in the right median cephalic vein for the intravenous (i.v.) injection of the autonomic blocking agents.

##### Squatting test

Each individual stood for 3 min (basal period), then squatted for 1 min (Sq) and then stood up again for 1 min (St). Blood pressure and HR were measured with the sphygmomanometer during the last minute of standing (basal HR) and immediately after the inception of squatting (HRSq) and standing (HRSt). HR was calculated from the average values of the RR intervals during cuff deflation. Thus, HRSq and HRSt reflected the average HR approximately 10–25 s after completing the squatting and standing manoeuvre, respectively. We calculated the difference between the basal HR and HRSq (ΔHRSq) and between HRSt and HRSq (ΔHRSt). All subjects were asked to perform the test in a completely relaxed manner without speaking.

##### Pharmacological autonomic blockade

Fifteen minutes after completing the control squatting test, a time at which the blood pressure, HR and ECG had returned to baseline levels, pharmacological autonomic blockade was induced by administering propranolol (0.2 mg/kg) over the course of 30 s. Immediately thereafter the second squatting test was started. After completing the second squatting test, the subjects returned to the supine position, atropine sulfate (0.04 mg/kg) was injected over the course of 30 s and participants then performed the third squatting test.

#### Study 2

The 51 patients with Type 2 diabetes mellitus underwent the squatting test and measurement of their BRS. The conditions were as in study 1.

##### Baroreflex sensitivity

With the patient in the supine position, we recorded arterial blood pressure continuously and non-invasively using a tonometer (Jentow-7700; Colin). The tonometric sensor was placed over the left radial artery. Arterial blood pressure and standard 12-lead ECG were monitored simultaneously and data were stored in a PCM data recorder (RD-200T; TEAC) for subsequent analysis. A venous catheter was placed in the right median cephalic vein for the i.v. injection of phenylephrine. BRS was assessed by the conventional phenylephrine method [13]; we injected 1–3 µg/kg phenylephrine over 5 s to obtain a systolic blood pressure increase of 15–40 mmHg. BRS was calculated as the slope of the linear regression line of the systolic blood pressure and the RR interval. Regression lines with a significant correlation coefficient (*r*) greater than 0.8 were used for subsequent analysis. The injection of phenylephrine was repeated twice and the mean of the two slopes was taken as the BRS value. According to previous studies [14,15], BRS was determined to be low when it was less than 3 ms/mmHg.

##### Squatting test

Twenty minutes after completion of the BRS study, a time at which the HR and blood pressure had returned to steady-state levels, the squatting test was started. We removed the tonometer and placed the sphygmomanometer over the left brachial artery. The protocol for the squatting test was as in study 1; ΔHRSq and ΔHRSt values were calculated for each patient.

### Statistical analysis

Data are presented as the mean ± SE. Student's *t*-test for paired observations was used to analyse changes produced by the manoeuvres. The correlation between two variables was assessed with the Pearson correlation coefficient. Correlation coefficients (*r*) were tested for statistical significance by means of Student's *t*-tests. A *P* value < 0.05 was considered statistically significant.

## Results

### Study 1—squatting test under autonomic blockade (healthy subjects):

**Figure 1:**
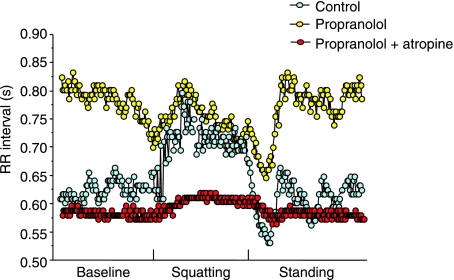
Example of RR interval changes during the squatting test before and after autonomic blockade in a healthy subject. In the control study, the RR interval lengthened immediately after squatting and shortened after standing. Propranolol suppressed a decrease in RR intervals during the baseline and standing periods. The additional administration of atropine suppressed an increase in RR intervals during squatting.

Figure 1shows a representative example of the RR interval changes during the squatting test before and after autonomic blockade in a 32-year-old healthy man. In the control study, the RR interval lengthened by a maximum of around 20 s immediately after the beginning of squatting and shortened by a minimum of around 15 s after the start of standing. After the injection of propranolol, the RR interval increased during all three manoeuvres. The difference in the RR interval was more pronounced during the baseline and standing manoeuvres than during the squatting manoeuvre. The additional administration of atropine (under the condition of complete autonomic blockade) resulted in marked blunting of the changes in the RR intervals during all three manoeuvres. Compared with the control values, the RR interval during squatting was markedly shortened after autonomic blockade.

Table 1 shows the average HR value in 20 healthy subjects during each manoeuvre (baseline, squatting, standing) of the squatting test before and after pharmacological autonomic blockade. In the control study, there was a significant difference between the baseline and the squatting manoeuvre; HR increased to above the baseline during the subsequent standing manoeuvre (*P* < 0.0001). After propranolol injection, HR was significantly lower during all three manoeuvres compared with the control values (*P* < 0.0001). After the additional injection of atropine, HR during all three manoeuvres was significantly higher than in the control study and in the study performed after the injection of propranolol (*P* < 0.0001).

**Table 1:** Heart rate responses to the squatting test before and after autonomic blockade in healthy subjects (*n* = 20)

	Baseline (beats/min)	Squatting (beats/min)	Standing (beats/min)
Control	81 ± 3	71 ± 2*	88 ± 2*†
Propranolol	66 ± 2‡	63 ± 2‡	72 ± 2‡
Propranolol + atropine	100 ± 2‡§	98 ± 2‡§	100 ± 2‡§

* *P* < 0.0001 vs. baseline;

† *P* < 0.0001 vs. squatting;

‡ *P* < 0.0001 vs. control values;

§ *P* < 0.0001 vs. after propranolol administration.

Our results suggest that both β-sympathetic and vagal activity are involved in the HR response during each manoeuvre of the squatting test. However, these HR responses might be controlled primarily by the parasympathetic- and the β-sympathetic tone during squatting and standing, respectively.

### Study 2—squatting test and BRS in diabetic patients:

Table 2 shows the average HR and blood pressure values recorded during the squatting test in 51 diabetic patients. HRSq was significantly lower than the basal HR; the HR increased significantly during the standing manoeuvre (*P* < 0.0001). There was a significant increase in the systolic and diastolic pressure when the patients assumed the squatting position after the baseline position (*P* < 0.0001). This was followed by a decrease when they stood up after squatting (*P* < 0.0001).

**Table 2:** Heart rate and blood pressure responses to the squatting test in diabetics (*n* = 51)

	Baseline	Squatting	Standing
Heart rate (beats/min)	81 ± 2	74 ± 2*	86 ± 2*†
Systolic blood pressure (mmHg)	118 ± 3	138 ± 3*	110 ± 2*†
Diastolic blood pressure (mmHg)	68 ± 1	78 ± 2*	63 ± 2*†

* *P* < 0.0001 vs. baseline

† *P* < 0.0001 vs. squatting.

**Figure 2:**
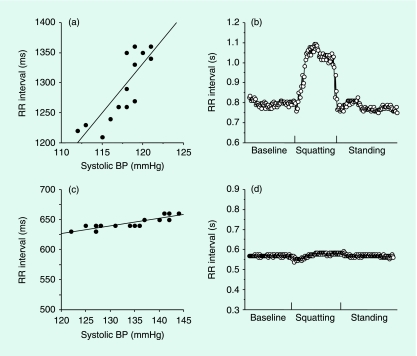
Baroreflex sensitivity (BRS) and RR interval changes during the squatting test. One 52-year-old diabetic man with normal BRS (14.1 ms/mmHg) showed remarkable changes in the RR interval during the squatting test (a, b). The other 52-year-old male diabetic patient who exhibited a very low BRS value (1.1 ms/mmHg) showed small RR interval changes during the squatting test (c, d).

**Figure 3:**
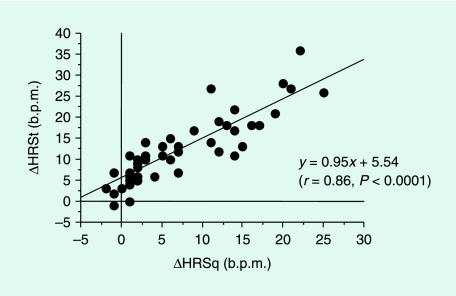
Correlation between ΔHRSq and ΔHRSt. ΔHRSq and ΔHRSt showed a significant positive correlation in all diabetic patients (*r* = 0.86, *P* < 0.0001).

One 52-year-old male diabetic patient with normal BRS (14.1 ms/mmHg) demonstrated remarkable RR interval changes during the squatting test (Fig. 2a and b). His ΔHRSq and ΔHRSt were 19 and 21 beats per min (b.p.m.), respectively. In contrast, another 52-year-old male diabetic patient whose BRS was very low (1.1 ms/mmHg) exhibited very minor RR interval changes during the squatting test (Fig. 2c and d). Both his ΔHRSq and ΔHRSt were extremely low (1 b.p.m.). Fig. 3shows a significant correlation between ΔHRSq and ΔHRSt in the 51 diabetic patients (*r* = 0.86, *P* < 0.0001).

**Figure 4:**
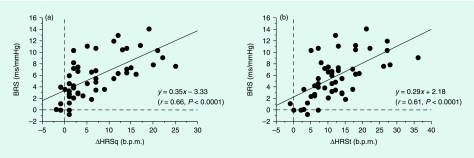
Correlation between squatting test results and baroreflex sensitivity (BRS). Both ΔHRSq (*r* = 0.66, *P* < 0.0001) and ΔHRSt (*r* = 0.61, *P* < 0.0001) showed a significant correlation with BRS in diabetic patients.

**Figure 5:**
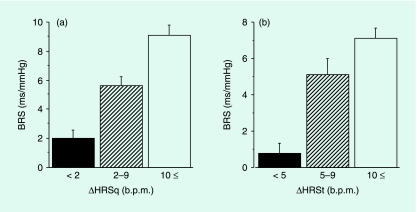
Baroreflex sensitivity (BRS) and squatting test results in diabetic patients. The diabetic patients were divided into three groups based on their ΔHRSq and ΔHRSt values. Patients with fewer than 2 b.p.m. at ΔHRSq or fewer than 5 b.p.m. at ΔHRSt had very low BRS values.

Figure 4shows that, in our diabetic patients, ΔHRSq and ΔHRSt were significantly correlated with BRS (*r* = 0.66, *P* < 0.0001 and *r* = 0.61, *P* < 0.0001, respectively). We divided all diabetic patients into three groups according to the ΔHRSq and ΔHRSt value. As shown in Fig. 5,patients with fewer than 2 b.p.m. of ΔHRSq or fewer than 5 b.p.m. of ΔHRSt exhibited a very low BRS value. In patients with more than 10 b.p.m. of ΔHRSq or ΔHRSt, the BRS value was almost normal. The sensitivity and specificity of ΔHRSq at fewer than 2 b.p.m. and at BRS lower than 3 ms/mmHg were 69 and 87%, respectively; at ΔHRSt of fewer than 5 b.p.m. and at BRS lower than 3 ms/mmHg, they were 46 and 97%, respectively.

The reproducibility of the squatting test indices was assessed as the sd of the differences between the first and second test; in terms of a per cent of the average value it was 7.1%; the correlation coefficient was 0.97 (*P* < 0.0001).

## Discussion

There is an established link between the autonomic nervous system and life-threatening arrhythmias and cardiovascular mortality. In diabetic patients, cardiac autonomic neuropathy must be identified early because it is closely associated with a high risk of cardiac mortality [1,2]. Quantitative approaches to assessing autonomic function are the determination of BRS [3–6] and HR variability [14,15] and ^123^I-metaiodobenzylguanidine (MIBG) scintigraphy [16,17]. In patients with myocardial infarction, heart failure and diabetes, BRS is of strong independent prognostic value [7,8,18–20]. The method most extensively used to determine BRS involves the bolus injection of the presser drug phenylephrine [13]. However, this method requires the i.v. administration of a pressor agent to raise the blood pressure and results in the artificial disruption of physiological cardiovascular function.

Previously, we proposed a new non-invasive method to assess BRS by downward tilting [9,10]. We studied the relationship between systolic blood pressure and RR interval changes during downward tilting and found a significant correlation between these parameters in healthy subjects [9] and diabetic patients [10]. BRS calculated by downward tilting also correlated significantly with BRS obtained with the conventional phenylephrine method. Although BRS determined by downward tilting elicited a greater physiological cardiovascular response than did BRS determined by the conventional method, its wide applicability is compromised by the need for an electrical tilt table and equipment for continuous blood pressure monitoring.

In the present study we used the squatting test, a simple and easily performed posture-change manoeuvre. The cardiovascular reflex seen in the posture change from standing to squatting was similar to that observed during downward tilting. The squatting test is a simple method to assess parasympathetic and sympathetic activity based on the HR response [11]. On the one hand, the immediate cardiovascular responses upon a posture change from standing to squatting consist of an increase in the blood pressure and RR intervals [21,22]. The rise in blood pressure in response to squatting is thought to be primarily as a result of an increase in venous return followed by an increase in cardiac output; this involves activation of arterial baroreceptors followed by the activation of vagal efferents, resulting in lengthening of the RR interval. That the reflex nature of squatting induces bradycardia is confirmed by pharmacological autonomic blockade studies that suggested mediation by the parasympathetic nerve. On the other hand, a posture change from squatting to standing resulted in a blood pressure decrease and RR interval shortening secondary to sympathetic nerve stimulation [23,24]. The present study also showed that tachycardia observed when the position changes from squatting to standing is primarily induced by sympathetic nerve stimulation coincident with vagal withdrawal.

According to Marfella *et al*. [11], the HR response observed after squatting and standing represents a simple, useful index to assess autonomic neuropathy in diabetic patients. They calculated their squatting test indices by using the basal RR interval (mean 10 beats just before squatting), the longest RR interval during the squatting manoeuvre and the shortest RR interval during the standing manoeuvre. We hypothesized that the HR change on altering the position from standing to squatting reflects the BRS value as well as the downward tilting method. As shown in Fig. 2, diabetic patients with severe autonomic dysfunction showed markedly depressed BRS values and little RR interval change during the squatting test; the values we obtained were similar to those shown by healthy volunteers during complete autonomic blockade (Fig. 1). Using the method of Marfella *et al*. [11] we previously demonstrated that measurements obtained with the squatting test correlated with BRS in diabetic subjects [12]. However, their method requires continuous ECG monitoring throughout the study to measure the beat-to-beat RR intervals and may limit the widespread use of the original squatting test in routine clinical practice. Therefore, we developed a simpler method to assess cardiovascular responses with the squatting test. We used a digital electronic sphygmomanometer, an instrument used widely to measure the blood pressure and HR automatically. Only 5 min are required to complete the squatting test and the presence of orthostatic hypotension can be determined at the same time.

We found that ΔHRSq significantly correlated with the BRS value. This is consistent with our previous findings based on downward tilting [10]. The arterial baroreflex influences both the sympathetic and parasympathetic limbs of the autonomic nervous system. ΔHRSq and ΔHRSt are primarily mediated by the parasympathetic and sympathetic nerve, respectively; however, both autonomic neurons are involved in both HR changes. Diabetic patients with autonomic dysfunction show reduced vagal tone combined with reduced ability to activate sympathetic tone [25]. Our data revealed a significant correlation between ΔHRSq and ΔHRSt; ΔHRSt also significantly correlated with BRS. In particular, diabetic patients with very low ΔHRSq (< 2 b.p.m.) or ΔHRSt (< 5 b.p.m.) values may be at high risk for cardiovascular death and require careful follow-up.

## Conclusions

We introduced a simple and easy method to assess cardiovascular autonomic tone with the squatting test. The new indices, ΔHRSq and ΔHRSt, showed a significant correlation with BRS. This method, which can assess BRS spontaneously, may provide a rational diagnostic alternative for identifying autonomic neuropathy in diabetic patients. Prospective studies involving a heterogeneous study population are underway in our laboratory to determine the prognostic significance of our squatting test indices and to confirm their clinical usefulness.

## Competing interests

Nothing to declare.

## Abbreviations

BRS: baroreflex sensitivity
ECG: electrocardiogram
HR: heart rate
HRSq: difference in heart rate between basal and squatting position
ΔHRSt: difference in heart rate between standing and squatting position
Sq: squatting
St: standing
